# Vulnerability, beliefs, treatments and economic burden of chronic obstructive pulmonary disease in rural areas in China: a cross-sectional study

**DOI:** 10.1186/1471-2458-12-287

**Published:** 2012-04-20

**Authors:** Peian Lou, Yanan Zhu, Peipei Chen, Pan Zhang, Jiaxi Yu, Ning Zhang, Na Chen, Lei Zhang, Hongmin Wu, Jing Zhao

**Affiliations:** 1 Xuzhou Center for Disease Control and Prevention, 142 West Erhuan Road, Xuzhou City, Xuzhou 221006, Jiangsu Province, People's Republic of China; 2 Department of Respiratory Medicine, Affiliated Hospital of Xuzhou Medical College, 99 West Huaiai Road, Xuzhou City, 221006, Jiangsu Province, People's Republic of China

**Keywords:** COPD, Vulnerability, Beliefs, Treatments, Economic burden

## Abstract

**Background:**

The incidence of chronic obstructive pulmonary disease (COPD) in China is very high. This study aimed to assess the vulnerability of COPD patients in rural areas outside Xuzhou City, Jiangsu province, in order to provide helpful guidance for future research and public policies.

**Methods:**

The vulnerability of 8,217 COPD patients was evaluated using a face-to-face questionnaire to obtain information on general characteristics, awareness, beliefs, medication usage, acute exacerbation of the disease, and economic burdens. Direct economic burdens were calculated based on the questionnaire, and indirect economic burdens were estimated using local per capita income and life expectancy in 2008. The years of potential life lost were calculated using loss of life years for each age group and multiplying by the number of deaths in a given age group.

**Results:**

Of the 8,217 patients, 7,921 (96.4%) had not heard of COPD, and 2,638 (32.1%) did not understand that smoking was a risk factor for COPD. No patients had used inhalers, nebulizer drugs or oxygen therapy, either regularly or sporadically. No patients had undergone pulmonary rehabilitation or surgical treatment, while 4,215 (51.3%) took theophylline to relieve dyspnea, and 3,418 (41.6%) used antibiotics to treat exacerbations. A total of 2,925 (35.6%) patients had been admitted to hospital during the past year because of respiratory symptoms. The average direct and indirect economic burdens on COPD patients were 1,090 and 20,605 yuan, respectively.

**Conclusions:**

The vulnerability of patients in rural Xuzhou to COPD was high. Their awareness of COPD was poor, their treatment during both the stable and acute exacerbation stages did not meet standards, and the economic burdens were large. Interventions are therefore needed to improve the prevention and management of COPD in this population. Further studies are required to verify these findings.

## Background

Chronic obstructive pulmonary disease (COPD) is a preventable and treatable disease [1,2], but it remains a significant public health problem. It is a major chronic cause of mortality and morbidity and has been identified as the third leading cause of mortality and morbidity worldwide [3-5]. The World Health Organization (WHO) estimated that 274 million people worldwide died of COPD in 2000 [1]. An estimated 43 million men and women in China have COPD [6], and the disease mortality is about 1.6% [7]. Management of patients with COPD must therefore become a priority in China.

COPD is characterized by airflow limitation that is not fully reversible, and a progressive decline in lung function [1]. Patients often exhibit chronic dyspnea and bronchitis, coughing, sputum production, and pathologic features of emphysema. COPD is often accompanied by exacerbation of respiratory symptoms requiring hospitalization [8]. The goals of managing stable COPD include reducing the frequency and severity of exacerbations, as well as controlling baseline symptoms [1,9,10].

The management of patients with COPD depends upon their understanding and recognition of the disease [11-13]; they then need to seek out a general practitioner who must also recognize the condition [14]. However, current management of COPD is primarily based on hospitalized cases in urban districts [15,16], and little attention has been paid to community-based cases in other areas.

Vulnerability refers to the susceptibility of a population to risks, reflected in the likelihood and extent of population health losses caused by certain factors or changes to the health-care system [17]. Vulnerability can influence people’s competence, increase their susceptibility to disease, and prolong recovery [18]. The cognitive vulnerability of COPD patients is affected by personal factors, social environment, medical history, and many other factors. Personal factors include a lack of knowledge of methods for the prevention and control of COPD, poor lifestyle, attitude, and high-risk behaviors. The social environment includes social support, availability of information, health care, economic aspects, and other factors. To evaluate the vulnerability of a population, it is necessary to identify the factors that lead to these aspects of vulnerability. Studies of the extent of cognitive vulnerability in patients with COPD can provide a scientific basis for developing targeted control measures.

This study therefore aimed to evaluate the vulnerability of patients with COPD, with respect to their comprehension of the disease, treatments and economic burdens, and to provide scientific evidence to improve the prevention and management of COPD in China and other countries.

## Methods

### Subjects

**Figure 1:**
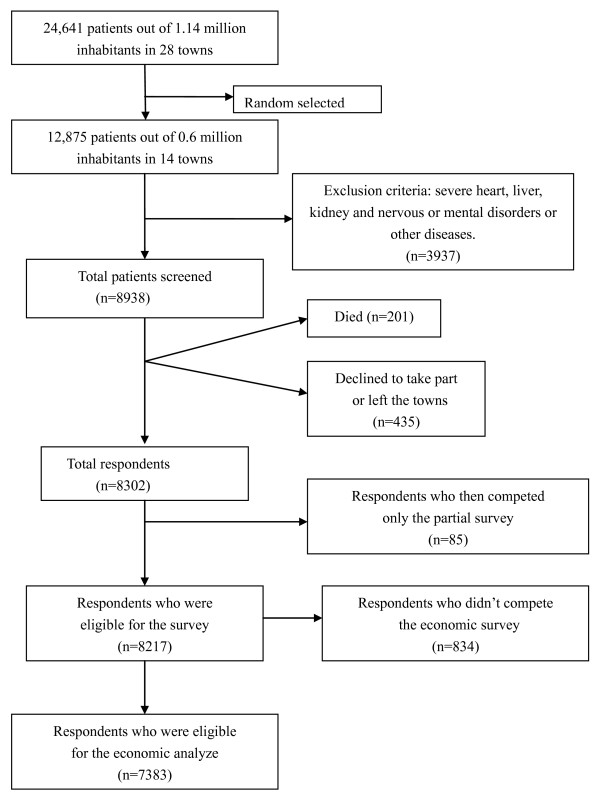
Sample construction.

Tongshan County, in the Xuzhou City region of Jiangsu province, has 28 townships and 1.14 million inhabitants. From a total of 1.10 million health records screened by the end of 2007, 24,641 cases of COPD were uncovered according to the COPD diagnosis and treatment guidelines criteria (revised 2007) [19]. The patients enrolled in the study are listed in Figure 1. According to the Global Initiative for Chronic Obstructive Lung Diseases (GOLD), COPD can be divided into levels I–IV [20].

### Questionnaire design

We designed a vulnerability questionnaire based on previously published domestic and foreign literature [21-23], which included information on aspects including epidemiology, prevention, treatment, and rehabilitation. The survey instrument consisted of 75 items and was divided into five domains: demographic data (10 items), cognition of COPD (15 items), behaviors (5 items), disease management (10 items), qualities of life (25 items) and economic burdens (10 items).

### Measurements of general data

The 8,217 patients with COPD were interviewed face-to-face in their homes. Information on age, gender, current employment status, level of education, cigarette-smoking status, marital status, physical activity, family history of COPD and other conditions were obtained using the designed questionnaire. The interview also obtained data on the cost of hospitalizations, outpatient costs, outpatient accommodation, in-home treatment costs, and the costs of long-term medication regimens including drugs purchased at the patient’s own expense, self-medication drugs, and costs related to lost productivity and lost employment in the last year (by examining the patient’s hospital records and records from township and village clinics). Hospital accommodation costs included the costs of patient transportation and meals, their escorts, and employment costs paid to caregivers.

### Calculation of economic burdens

Direct economic burdens were calculated using the questionnaire. Indirect economic burdens were estimated using local per capita income and life expectancy for 2008. In order to estimate the risk of premature death, the number of years of potential life lost (YPLL) as a result of COPD per 100,000 people was assessed. According to the local life expectancy statistics, the reference survival age was set at 70 years, and the loss of potential life was defined as between 70 years and the age of the patient at death. The age composition of China's population census report of 2000 was used to adjust the composition of the population in 2008 in Tongshan County. The YPLL was calculated as follows: YPLL = Σ (ai × di), where ai = loss of life years for a certain age group, and di = the number of deaths in a given age group. Average YPLL = YPLL/deaths [24].

This study was carried out between March 2008 and March 2009 in the rural area surrounding Xuzhou City in Jiangsu province, China. The study was approved by the Ethics Committee of the Xuzhou Center for Disease Control and Prevention, and the Regional Ethical Vetting Board, Xuzhou, China. Informed consent (verbal and written in English, translated to Igbo when necessary) was obtained from all participants.

### Statistical analysis

Data analysis was performed using the Statistical Package for Social Science (SPSS) version 13.0. Numerical variables were expressed as means ± standard deviations (SD). For categorical variables, the percentages of patients were calculated.

## Results

### General characteristics

A total of 8,302 questionnaires were sent out and all were returned. Of these, 8,217 were complete and valid, giving a response rate/compliance rate of 99.0%. There were 3,930 male patients and 4,287 female patients (Table 1). Patient ages ranged from 36–84 years (mean ± SD, 61.3 ± 14.3). Patients between 55 and 75 years of age accounted for 63.4% of the total. According to the GOLD classification criteria, 1,216 patients with COPD were classified as stage I (14.8%), 3,435 as stageII(41.8%), 2,161 as stage III (26.3%), and 1,405 as stage IV (17.1%).

**Table 1:** Basic feature of COPD patients

***Variable***	***N***	***(%)***
Sex		
male	3,930	47.8
female	4,287	52.2
Age (years)		
<40	620	7.6
40~	2,566	31.2
60~	4,297	52.3
80~	734	8.9
Marital status		
unmarried	172	2.1
married	5,890	71.7
divorced	43	0.5
widowed	2,112	25.7
Living arrangement		
live alone	1,018	12.4
live with spouse	4,547	55.3
live with children	2,315	28.2
Others	337	4.1
Education		
illiterate	4,665	56.8
primary school	1739	21.2
junior school	1,090	13.3
high school	723	8.8
Household income per month (yuan)		
<300	3,541	43.1
300~	2,337	28.4
500~	1,613	19.6
1,000~	726	8.8
Disease history (years)		
<5	796	9.7
5~	1,048	12.8
10~	1,928	23.5
20~	3,903	47.5
30~	542	6.6

### Cognitive vulnerability

Among all the patients, 7,921 (96.4%) had never heard the term ‘COPD’, and most of them thought they had contracted chronic bronchitis or bronchial asthma. The cognitive vulnerabilities are shown in Table 2. Although most patients were concerned about their health, 6,163 (75.0%), had taken no appropriate measures, such as preventing cold, maintaining a regular vaccination schedule or taking globulin injections. However, 93.9% of patients said that they were willing to change their behaviors (including quitting smoking and undergoing rehabilitation or physical training) to delay development of the disease. Only a very small number of patients had no opinion or were unwilling to change their unhealthy behaviors.

**Table 2:** Cognitive vulnerability of patients with COPD

***Variable***	***N***	***(%)***
Never heard the term ‘COPD’	7,921	96.4
Told they had the following diseases, rather than COPD		
chronic bronchitis	6,541	79.6
emphysema	1,241	15.1
bronchial asthma	444	5.4
Thought their respiratory symptoms were related to the following factors		
cold	7,240	88.1
smoking	4,150	50.5
passive smoking	3,106	37.8
air pollution	3,878	47.2
exposure to cold air and change in climate	7,691	93.6
Believed their conditions were due to after effects of the following diseases		
chronic bronchitis	3,920	47.7
asthma	3,500	42.6
not knowing smoking was the main risk factor	2,638	32.1
had taken no appropriate measures	6,163	75.0
willing to change their bad behaviors	7,716	93.9

### Vulnerability to disease management

The local health administration had not implemented any health-education programs for COPD patients, such as quitting smoking and maintaining a reasonable diet. Of the total, 649 (7.9%) patients did not wish to accept treatment because they thought their condition was incurable, while 6,853 (83.4%) patients wanted to accept treatment but were unable to continue to do so for financial reasons. None of the patients had used inhalers, nebulizer drugs or oxygen therapy, either regularly or sporadically. None of the patients had undergone pulmonary rehabilitation or surgical treatment, while 4,215 (51.3%) patients took theophylline to relieve their dyspnea, and 3,418 (41.6%) used antibiotics to treat exacerbation. Most patients underwent treatment in the village or township hospitals, but this treatment was informal and spontaneous. A total of 2,925 (35.6%) patients had received in-patient treatment in township hospitals due to respiratory symptoms within the past year.

### Economic vulnerability

The mean monthly per capita income of patients with COPD was (300 ± 165) yuan, while that of 3,541 (43.1%) patients was below 300 yuan. The yearly net per capita income of a farmer in Tongshan County was 6,340 yuan in 2008, or 528 yuan per month. The monthly per capita income of patients with COPD was 34.1% lower than that of the general population. COPD patients were unable to work for a mean of 150 days per year, while family members were prevented from working for a mean of 59 days per year. The median direct economic burden of patients with COPD in the rural areas in Tongshan County was 1,090 yuan; the medical costs of the 7,383 patients were 14,042,466 yuan, and the per capita economic burden was 1,902 yuan. The cost of outpatient services was 5,939,963 yuan, accounting for 42.3% of the total. This was followed by hospitalization costs (4,563,801 yuan, 32.5%) and long-term self-financed medicines (2,878,710 yuan, 20.5%). These three categories accounted for 95.3% of COPD-related medical costs. The mean yearly per capita income of families of COPD patients was 3,600 yuan, and the mean yearly direct economic burden of one COPD patient was 1,090 yuan. COPD therefore accounted for an average of one third of the family income. Regarding the indirect economic burden, the total YPLL of patients living in Tongshan County in 2008 was 682.5 years, with an average of 3.25 years per patient, calculated using the formula, YPLL = Σ (ai × di). The total number of years lost by men was 427.5, with an average of 1.76 per person. The number of years lost by women was 255 years, with an average of 1.18 per person. Taking into account Tongshan farmers’ yearly net per capita income (6,340 yuan) in 2008, the indirect economic loss caused by COPD was estimated at 4,327,050 yuan in total, and 20,605 yuan per capita.

## Discussion

This is the first study to investigate the vulnerabilities caused by COPD in China. The results indicated high cognitive vulnerability of COPD patients in rural areas of Xuzhou. Although health education is the most effective means of improving symptoms in patients with COPD [25], no health education programs were implemented in the current survey region. Most of the patients (96.4%) in the current study had never heard the term ‘COPD’ before, which was fewer than reported by Walker *et al.*[26]. Lung function tests and health education programs were not implemented in these patients either. These results were also consistent with the findings published by Ning *et al.*[27]. Tobacco smoking is the best-known causal factor associated with the development of COPD, and cessation of smoking is the only measure that can prevent the disease and modify its clinical course [28]; however, our results showed that 32.1% of patients did not know that smoking was the main risk factor for COPD. The health education system thus needs to be improved in order to raise the awareness of patients to COPD.

Medication can effectively prevent and control the symptoms of COPD, reduce the frequency and severity of exacerbations, and improve both exercise tolerance and quality of life [29,30]. Optimizing management of COPD patients, especially stable patients, can reduce the frequency of acute episodes [31]. Our survey showed that patients in rural Xuzhou were highly vulnerable to poor disease management. None of the stable patients received regular drug treatment, and the drug-regimen-compliance rate was significantly lower than that reported by Barr *et al.*[32]. No individuals used inhalers or spray treatments during the acute exacerbation stage of COPD, which is well below the normal rate of inhaler use of approximately 10%, reported by Restrepo *et al.*[33]. There has been a recent increase in interest in pulmonary rehabilitation programs, in line with clinical evidence clearly demonstrating reduced dyspnea, increased exercise tolerance, improved physical and emotional participation, and decreased health-care costs [34]. The current results showed that none of the patients in rural Xuzhou understood how to perform rehabilitation exercises. Influenza vaccination has been shown to prevent acute respiratory infections in patients with all severities of COPD [35], and influenza and pneumococcal vaccinations are recommended as an important risk-reduction strategy [36]. Additionally, mainly for financial reasons, patients in the current study were only hospitalized in rural hospitals, which did not provide health education.

The yearly per capita net income of Tongshan rural farmers in 2008 was 6,340 yuan, or 528 yuan per month, but the monthly per capita net income of COPD patients was 34.1% lower. The average annual income of COPD patients’ families was 3,600 yuan, which was 43.2% lower than that of rural farmers. In addition, COPD patients experienced an annual direct economic burden of 1,090 yuan as a result of medical expenses, accounting for one third of their families’ incomes. COPD caused a potential loss of 3.25 years of life per patient, which was higher than that reported by Fei *et al.*[36]. The indirect loss of income was calculated to be as high as 20,605 yuan per capita, indicating that COPD had a detrimental effect on the local population. In addition to more measurable losses, many patients lost their ability to work. This direct economic burden was less than that reported for developed countries, such as Spain, the United States and many European countries [37,38]. However, the annual per-capita income in these countries is also much higher than in rural China, and the COPD patients in this study might thus suffer a larger direct economic burden than patients in the U.S. and Europe.

This study was limited by focusing primarily on farmers, who do not have fixed working hours. This made it difficult to calculate accurately the number of working days lost as a result of disease. Patients also lacked formal treatment, and it was therefore difficult to obtain reliable information on medical economics.

## Conclusions

Patients with COPD experienced greater cognitive vulnerability, poorer disease management, and greater disease burden than healthy people. Interventions are needed to improve the prevention and management of COPD. Further studies are needed to verify these findings.

## Competing interests

The authors declare that they have no competing interests.

## Authors' contributions

PL participated in writing the title and abstract, reviewed the text, and contributed to writing the manuscript. YN conceived the study, participated in the study design, writing the title and abstract, and editing the text, as well as in data extraction and analysis and drafting of the manuscript. PC performed literature searches, participated in writing the title and abstract and reviewing the text, and contributed to the manuscript drafts. PZ and JY conceived the study, participated in the study design and in writing the title and abstract, as well as editing the text and contributing to the manuscript drafts. NZ and NC contributed to the conception of the study, participated in the study design, and contributed to the manuscript drafts. LZ, HW and JZ were the lead authors of the original review, contributed to the conception of the study, participated in the study design, and contributed to the manuscript drafts. All authors read and approved the final manuscript.

## Pre-publication history

The pre-publication history for this paper can be accessed here:

http://www.biomedcentral.com/1471-2458/12/287/prepub
